# Reliability, validity, and sensitivity of short-form 36 health survey (SF-36) in patients with sick sinus syndrome

**DOI:** 10.1097/MD.0000000000033979

**Published:** 2023-06-16

**Authors:** Qiaomin Wu, Yao Chen, YuTong Zhou, Xin’ai Zhang, Yu Huang, Ruxiu Liu

**Affiliations:** a Guang’anmen Hospital, China Academy of Chinese Medical Sciences, Beijing, China.

**Keywords:** reliability, sensitivity, short-form 36 health survey, sick sinus syndrome, validity

## Abstract

Patients with sick sinus syndrome (SSS) experience a decrease in health-related quality of life (HRQoL), but there is currently no scale available to measure their unpleasant symptoms. The Short Form 36 Health Survey (SF-36) is a commonly used scale to assess HRQoL. In this study, we aimed to evaluate the reliability, validity, and sensitivity of SF-36 in patients with SSS. The sample included 199 eligible participants. We estimated the reliability through test-retest reliability, internal consistency, and split-half reliability. To examine the validity of the questionnaire, confirmatory factor analysis, convergent validity, and discriminant validity were conducted. Sensitivity was determined by the differences in age (cutoff 65 years) and New York Heart Association class. The intraclass correlational coefficients scores showed high test-retest reliability (intraclass correlational coefficients > 0.7). The overall Cronbach α was 0.87 (8 scales range: 0.85–0.87), showing good internal consistency reliability. The split-half reliability coefficient of the SF-36 is 0.814, indicating good reliability. Factor analysis showed that SF-36 subscales could be drawn into 6 components that explain 61% of the total variance. Results of model fit indicate comparative fit index = 0.9, incremental fit index = 0.92, Turker–Lewis index = 0.90, approximate root mean square error = 0.07, and normalized root mean square residual = 0.06. Convergent validity and discriminative validity showed adequate results. Comparison of different ages and New York Heart Association class groups showed statistical significance on most SF-36 subscales. We confirmed the SF-36 as a valid instrument for evaluating HRQoL patients with SSS. The reliability, validity, and sensitivity of SF-36 are acceptable for patients with SSS.

## 1. Introduction

Health-related quality of life (HRQoL) is a multidimensional concept derived from quality of life (QOL), covering the physical, psychological and social dimensions of health. Biomedical outcomes, not quality of life, have long been the focus of medical and health-related research. However, in recent decades, many studies have evaluated quality of life measures, particularly in relation to disorders and syndromes such as depression, obesity, disability, cancer, and fatigue.^[1]^ HRQoL data provide the evidence base for clinical decision-making and preventive medicine, promoting better patient-centered solutions for health and well-being guided by evidence-based treatment, psychosocial interventions, doctor-patient communication, and resource utilization. Thus, QOL has been considered a strong survival predictor, carrying prognostic importance.^[2]^

Sick sinus syndrome is a complex cardiac arrhythmia that is the primary indication for permanent pacemaker implantation in more than 50% of patients.^[3]^ It is characterized by bradycardia, sinoatrial block, sinoatrial pauses, atrioventricular block, and tachyarrhythmias and classic symptoms include syncope, decreased performance, and fatigue.^[4]^ Bradyarrhythmia therapy relies on the implantation of the electronic pacemaker. With deeper research, some researchers hold the view that pacemaker support life but do not heal the heart.^[5]^ In general, arrhythmias are relevant to lowered quality of life and uncertainty, causing patients to lack confidence in the treatment decision options and self-management. Thus, patients self-reporting may help them identify their issues, and understand the disease and its consequences.

In the medical area, HRQoL has been defined and evaluated in a multitude of ways. The short form 36 (SF-36) is the most widely used and extensively validated HRQoL measure. The questionnaire is composed of 36 items that can be summarized into 8 areas: Physical function (PF), which assesses whether health conditions interfere with normal physical capacity; Physical role (RP), which measures functional limitations due to health problems; Bodily pain, which measures degrees of pain and affection to day-to-day activities; General health (GH), which evaluates individual health status and its development tendency; Vitality (VT), which is a subjective assessment of energy and tiredness; Social function (SF), which impacts in quantity and quality of social activities induced by mental and physical problems; Role emotional (RE), which evaluates functional limitations by emotional problems; Mental health (MH), which measured aspects of depressive and anxiety. The 8 subscales can be further divided into physical component summary (PCS) and mental component summary (MCS). The SF-36 has shown good physiological and psychometric properties in a variety of patient groups and with normative data available.

We conducted a search on PubMed using the strategy (“questionnaires” OR “outcome measures”) AND (“arrhythmia” OR “bradycardia” OR “sick sinus syndrome”) AND (quality of life) to identify studies on arrhythmia-related quality of life. Out of 299 articles, we found 14 that examined the quality of life of patients after pacemaker implantation using various questionnaires such as SF-36, SF-12, and MLWHF. However, these studies did not evaluate the validity and reliability of the SF-36 scale in these patients. While previous research has demonstrated good validity and reliability of the SF-36 in assessing the quality of life in patients with coronary heart disease,^[6]^ there is a lack of such evaluation in patients with sick sinus syndrome. Given the importance of understanding outcomes across age groups and disease stages from HRQoL in clinical decision-making, further investigation into HRQoL in sick sinus syndrome (SSS) patients is warranted.

As our population grows and ages, the total number of SSS patients continues to rise. Understanding the association of sick sinus syndrome with HRQOL can help physicians provide more patient-centered therapy recommendations. The primary objective of this study was to evaluate the mental and physical health of SF-36 in patients with SSS in a clinical setting. This study will help assess HRQOL in patients with SSS and expand the use of SF-36.

## 2. Methods

### 2.1. Subjects

The sample size was calculated as 5 to 10 times the number of SF-36 items, the minimum sample size necessary for the current study was 180.^[7]^ Patients from the Guang’anmen Hospital, China Academy of Chinese Medical Sciences, were considered eligible for inclusion in the study if they spoke Chinese, expressed themselves clearly, thought clearly, and had been medically diagnosed with SSS (according to the 2012 ACCF/AHA/HRS guidelines^[8]^). Data were collected from September 2018 through February 2022. We collected information about sociodemographic characteristics, such as age, gender, educational level, marital status, and duration of SSS. Each participant was asked to complete the questionnaires by themselves under general conditions. This research was approved by the ethics committee of Guang’anmen Hospital (NO.2018-105-KY-01) and was conducted in accordance with the principles of the Helsinki Declaration.

### 2.2. Instrument

The SF-36 was translated into Chinese by the Zhejiang University School of Medicine.^[9]^ Thirty-six questions consisted that can be divided into 8 dimensions. The scores of SF-36 were converted to norm-based scoring, the mean score of the norms is 50, and the standard deviation is 10.^[10]^ Higher scores are associated with better HRQOL. The norm-based scores allow for comparing scores between different populations or health conditions.

### 2.3. Floor and ceiling effects

The floor or ceiling effect occurs when respondents score near the lower or upper limit on questionnaires, which possibly illustrates that their health status has deteriorated or improved, respectively. In this study, the percentage of patients with floor or ceiling effects was calculated. A value above 20% is considered to indicate a high level.^[11]^

### 2.4. Reliability

Reliability was examined in terms of test-retest reliability, internal consistency, and binary reliability. Two-week test-retest reliability was assessed by calculating intraclass correlational coefficients (ICC). ICC values >0.7 are generally considered as indicative of statistically reproducible results.^[12]^ Internal consistency of the SF-36 scale was evaluated by Cronbach alpha, which assesses the relationship between multiple items on the test and measures whether items are measuring the same construct. A Cronbach alpha value above 0.7 is often seen as a sign of satisfactory internal consistency and reliability.^[13]^ Binary reliability, which is also a kind of internal consistency reliability coefficient, was measured through the split-half item scores. Items of the SF-36 were split into 2 portions according to odd and even numbers, after which items were evaluated by the Guttman split-half coefficient. A correlation coefficient of 0.7 between 2 variables indicates a significant and positive relationship.

### 2.5. Validity

To examine structural validity of the questionnaire, confirmatory factor analysis (CFA) were conducted. SF-36 data were tested by Kaiser–Meyer–Olkin and the Bartlett sphere to examine the suitability for factor analysis. The number of factors was determined by the scree plot and parallel analysis.^[14]^ The fit of the model was evaluated by the comparative fit index (CFI), the incremental fit index (IFI), the Turker-Lewis index (TLI), the approximate root mean square error (RMSEA) and the normalized root mean square residual (SRMR). The CFI, IFI, and TLI exceed 0.9, and RMSEA and SRMR below 0.08 indicate adequate fit in the factor model.^[15]^

To assess the convergent validity, we calculated the composite reliability (CR) and average variance extracted (AVE). The CR is considered acceptable if it is >0.70, and the AVE is considered acceptable if it is >0.5.

Discriminating validity reflects the ability of a measurement instrument to distinguish between 2 related but distinct concepts or constructs. Discriminating validity was assessed by comparing the squared AVE and correlation between 2 constructs.^[16]^ According to previous research,^[17]^ the square root of AVE for each factor should be greater than the correlation between that factor and the other factors in the model.

### 2.6. Correlations and sensitivity

The correlation between SF-36 norm-based subscale scores and related characteristics (such as age, duration of the disease, and syncope) was analyzed with the Pearson product-moment correlation coefficient. In addition, SF-36 subscale scores of patients were analyzed in 2 aspects: groups demarcated at 65 years and New York Heart Association (NYHA) symptom class II/III. Sensitivity was assessed by effect size (ES) using Cohen d. ES informs about the strength of the relationship between 2 sets of variables or groups through dividing the difference between the means of 2 groups by SD. Based on Cohen definition, ES values of 0.2, 0.5, and above 0.8 were classified as “small,” “medium” and “large.”

## 3. Statistical analysis

Data were double-inputted and checked in a database using EpiData 3.1 software (EpiData Association, Odense, Denmark). R software (version 4.1.0) for statistical analysis. *P* values of <.05 (2-sided) were set as statistically significant.

We performed descriptive statistics to assess participant characteristics and the distribution of subscale scores based on the SF-36 norm. Categorical variables were analyzed using nonparametric statistical analysis. Although data with non-normal distribution are generally analyzed using nonparametric methods, the norm scores of SF-36 provided only means and SD. Therefore, we used parametric analyses for the SF-36 scores, despite potential deviations from normality in the distributions.

## 4. Results

### 4.1. Subjects

A total of 199 SSS patients were invited to participate. Table 1 shows the demographic and clinical characteristics of the patients. The mean age of the patients was 63.08 ± 11.59 years, 38.7% were men, 84.9% had bradycardia, and 56.8% had sinus arrest.

**Table 1:** Baseline characteristics of the Chinese patients with sick sinus syndrome (N = 199).

Characteristics	Mean (SD)/Number (%)
Age (yr)	63.08 (11.59)
Gender (male/female)	77 (38.7)/122 (61.30)
Martial status	
Married	184 (92.5)
Others	15 (7.5)
Educational level	
Elementary school and below	29 (14.6)
Junior and senior high school	112 (56.6)
College and above	57 (28.8)
Disease duration (yr)	7.61 (8.41)
Diagnose age (yr)	56.02 (12.59)
Bradycardia (yes/no)	169 (84.9)/ 30 (15.1)
Sinoatrial block (yes/no)	34 (17.1)/ 165 (82.9)
Atrioventricular block (yes/no)	17 (8.5)/ 182 (91.5)
Bundle branch block (yes/no)	8 (4)/ 191 (96)
Sinus arrest (yes/no)	113 (56.8)/ 86 (43.2)
Syncope (yes/no)	57 (28.6)/ 142 (71.4)
NYHA (II/III)	181 (91.0)/ 18 (9.0)

NYHA = New York Heart Association, SD = standard deviation.

### 4.2. Score distribution

Compared to norms, SSS patients had lower scores (<50) on SF-36, except for VT, SF, and MCS. Table 2 summarizes the numbers and proportions of individuals who occurred the floor and ceiling values in the SF-36. The floor effect of the SF-36 subscale was high in RP (39%), and the ceiling effect was observed in SF (33.17%). Most SF-36 values were free of floor and ceiling effects, ranging from 0% to 18% and 0% to 18.59%, respectively.

**Table 2:** Distribution of scores and floor/ceiling effects of the SF-36 assessed among the Chinese patients with SSS at baseline.

SF-36 subscale	Item	Mean	SD	Floor/ceiling effect (n, %) * at present study	
				Floor effect	Ceiling effect
PF	10	45.28	9.29	0 (0)	9 (4.52)
RP	4	33.77	22.12	77 (0.39)	0 (0)
BP	2	46.54	7.05	0 (0)	36 (18.09)
GH	5	36.92	11.25	1 (0.01)	0 (0)
VT	4	51.75	9.17	0 (0)	37 (18.59)
SF	2	53.44	10.41	0 (0)	66 (33.17)
RE	3	43.63	22.57	36 (0.18)	0 (0)
MH	5	49.94	9.93	0 (0)	19 (9.55)
PCS		40.15	10.38		
MCS		51.86	13.5		

BP = bodily pain, GH = general health, MH = mental health, MCS = mental component summary, PF = physical function, PCS = physical component summary, RE = role emotional, SF = social function, SF-36 = Short Form 36, SSS = sick sinus syndrome, RP = physical role, VT = vitality.

* Floor and ceiling effect (n, %): the numbers and percentages of the patients diagnosed of SSS with demonstrating the lowest and highest possible scores.

### 4.3. Reliability

The ICC scores showed high test-retest reliability (ICC > 0.7). The Cronbach alpha coefficient of the total SF-36 was 0.87, and the 8 subscales ranged from 0.85 to 0.87, all above the recommended value of 0.7. The results showed that SF-36 subscale scores had good internal consistency in SSS. The split-half reliability coefficient of the SF-36 was 0.814, indicating good reliability.

We observed that all correlation coefficients among subscales in the SF-36 were <0.70, except for SF to VT (0.73) and MH to VT (0.77). Subscales with similar constructs, such as MH and VT, had higher coefficients between scales (0.77). PF had a close correlation with GH, VT, SF, and MH (>0.5) (see Table 3).

**Table 3:** Internal consistency reliability and correlation among subscales in SF-36 (N = 199).

SF-36 subscale	ICC	Cronbach’α	PF	RP	BP	GH	VT	SF	RE	MH
PF	0.816	0.86	1							
RP	0.756	0.86	0.48	1						
BP	0.827	0.87	0.31	0.29	1					
GH	0.754	0.86	0.51	0.38	0.3	1				
VT	0.805	0.85	0.6	0.46	0.31	0.47	1			
SF	0.818	0.85	0.66	0.49	0.37	0.5	0.73	1		
RE	0.714	0.86	0.44	0.57	0.21	0.32	0.46	0.55	1	
MH	0.851	0.86	0.54	0.32	0.27	0.34	0.77	0.68	0.54	1

BP = bodily pain, GH = general health, ICC = intraclass correlational coefficients, MH = mental health, PF = physical function, RE = role emotional, RP = physical role, SSS = sick sinus syndrome, SF-36 = short form 36, SF = social function, VT = vitality.

### 4.4. Validity

**Figure 1.**
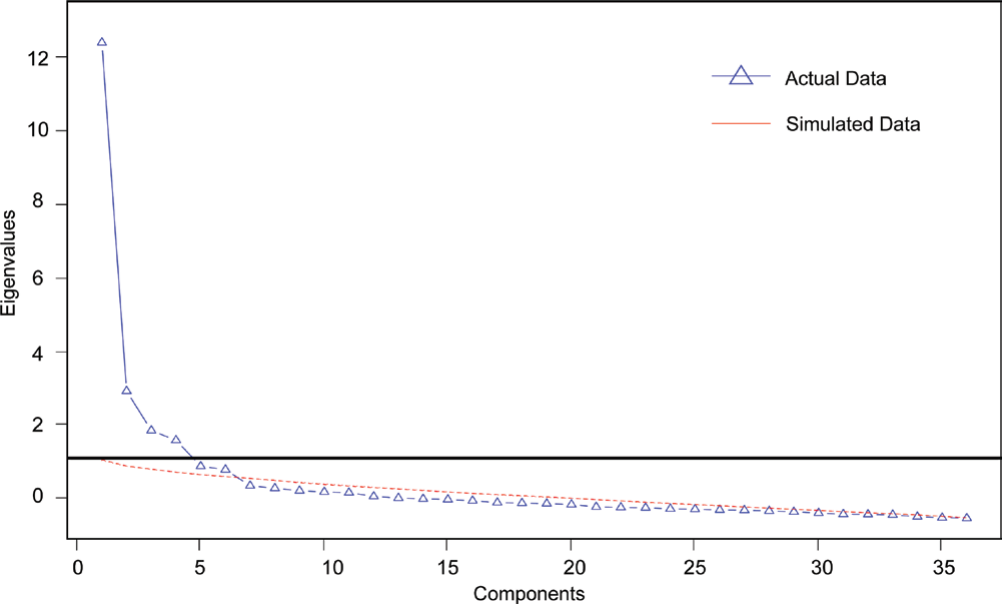
Parallel analysis scree plots.

The Kaiser–Meyer–Olkin value was 0.91 (>0.60), and the chi-square value of the Bartlett sphere test was 5178.872 with statistical significance (*P* < .001). These data were feasible for factorial validity. Figure 1 reveals that SF-36 subscale scores could be drawn into 6 common components via scree plot, explaining 61% of the total variance by PCA. Results of the model fit indicate CFI = 0.91 (> 0.9), IFI = 0.92 (> 0.90), TLI = 0.90 (= 0.90), RMSEA = 0.07 (< 0.080), and SRMR = 0.06 (< 0.08). Results were in acceptable ranges, indicating that the 6 factors were validated. The first factor covered MH, VT, and SF, explaining 29% of the variance. The second factor, RP, explains 19% of the variance.

**Figure 2.**
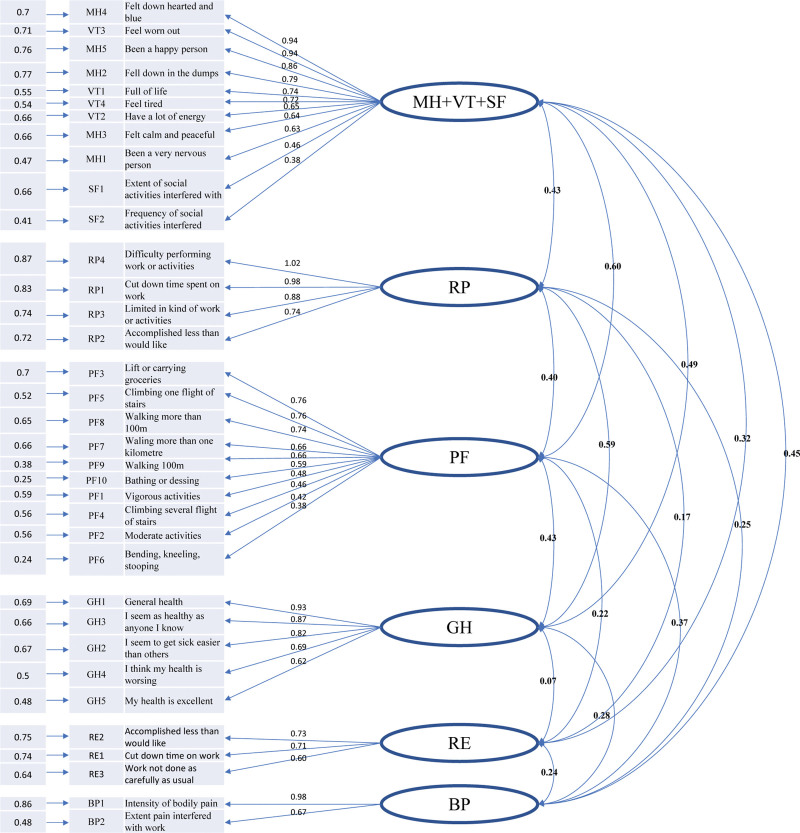
CFA analysis for the construct validity of SF-36 items. BP = bodily pain, CFA = confirmatory factor analysis, GH = general health, MH = mental health, PF = physical function, RE = role emotional, RP = physical role, SF = social function, SF-36 = short form 36, VT = vitality.

To better understand the construct validity of SF-36 for patients with SSS, the CFA results and standardized estimation for the adjusted model are presented in Figure 2. The effect of “Difficulty performing work or activities” strongly affects RP, with standardized coefficients of 1.02. The poorest loading factors are “Frequency of social activities interfered” to factor 1 (MH + VT + SF) and “Bending, kneeling, stooping” to PF, both with standardized coefficients of 0.38. Most item-scale correlation coefficients are beyond 0.40, reaching satisfactory convergent validity. Relationships among the 6 factors are also present in Figure 2. The results showed that the correlations between factor 1 (MH + VT + SF) and PF are higher than those between other factors across the PCS and MCS parts. GH with RE has the poorest relationship across the PCS and MCS parts. RP with GH is the most strongly related in the PCS part. Factor 1, which covers MH, VT, and SF, shows a close link between them in the MCS parts, and factor 1 to RE in the MCS parts has standardized coefficients of 0.32.

For convergent validity, the results of this study indicate adequate convergent validity as the AVE was above 0.50 except for PF and RE. CR was above 0.70 for all factors. Table 4 shows the square root of each AVE in the italicized diagonal, which is greater than the correlation score for other latent variables. The results indicate that the measurement model confirmed good convergent validity and discriminant validity.

**Table 4:** Convergent and discriminant validity results.

Factors	CR	AVE	MH + VT + SF	RP	PF	GH	RE	BP
MH + VT + SF	0.92	0.53	0.73					
RP	0.95	0.83	0.43	0.91				
PF	0.85	0.37	0.6	0.4	0.61			
GH	0.82	0.55	0.49	0.59	0.43	0.74		
RE	0.72	0.47	0.32	0	0.22	-0.05	0.69	
BP	0.82	0.70	0.45	0.25	0.37	0.28	0.24	0.84

The italicized diagonal shows the square root of each latent variable.

AVE = the average variance extracted, BP = bodily pain, CR = composite reliability, GH = general health, MH = mental health, PF = physical function, RP = physical role, RE = role emotional, SF = social function, VT = vitality.

### 4.5. Associations between SF-36 and disease-related characteristics

Pearson product-moment correlation coefficients between SF-36 subscale norm-based scores and clinical and demographic variables are summarized in Table 5. The SF-36 subscale scores were correlated with the age group in PF, RP, GH, and PCS. Meanwhile, there were significant inverse correlations between SF-36 subscale scores and the NYHA group in PF, VT, SF, and MH. However, there were no significant correlations between SF-36 subscale scores and disease duration or syncope episodes.

**Table 5:** Pearson product-moment correlation coefficient (Pearson r) between the norm-based SF-36 subscale scores and characteristics among patients with sick sinus syndrome (N = 199).

SF-36 subscale	Age	Disease duration	Syncope	NYHA
PF	−0.33 *	−0.13	−0.22	−0.25 *
RP	−0.29 *	−0.07	−0.1	−0.18
BP	−0.17	−0.15	0.02	−0.09
GH	−0.24 *	−0.02	−0.14	−0.15
VT	−0.13	−0.07	−0.1	−0.33 *
SF	−0.2	−0.06	−0.12	−0.31 *
RE	−0.16	−0.09	−0.09	−0.1
MH	0.01	−0.1	−0.15	−0.28 *
PCS	−0.39 *	−0.09	−0.12	−0.19
MCS	−0.03	−0.07	−0.1	−0.19

* *P* < .05 assessed by the Pearson product-moment correlation coefficient.

BP = bodily pain, GH = general health, MH = mental health, MCS = mental component summary, NYHA = New York Heart Association, PF = physical function, PCS = physical component summary, RE = role emotional, RP = physical role, SF-36 = short form 36, SF = social function, VT = vitality.

We also assessed sensitivity in the age and NYHA groups (see Table 6–7). In the age group, moderate sensitivity was found in the PF (ES = 0.64), RP (ES = 0.52), and PCS (ES = 0.57). NYHA group showed a large degree of responsiveness in the PF (ES = 0.9), VT (ES = 1.22), SF (ES = 1.14), and MH (ES = 1.04).

## 5. Discussion

We evaluated HRQoL by SF-36 among Chinese patients with SSS cross-sectionally in the present study. The main results achieved in the present study were as follows: We validated the reliability and validity of SF-36 among Chinese SSS patients; The SF-36 subscale scores focused on the physical health subscales among SSS patients were worse than the general population; Patients with aging and decreased activity showed a decline in the SF-36 subscale scores.

Our study provides evidence supporting the acceptable reliability of SF-36 among Chinese patients with SSS, as indicated by satisfactory internal consistency. However, we observed high ceiling effects in the SF subscale and potential floor effects in RP, which may limit the sensitivity of the instrument in evaluating patients with SSS. Notably, our findings suggest that middle-aged and older individuals in China have increasingly engaged in group activities, possibly reflecting a cultural shift toward valuing social connections later in life. The SF scores were lower than 50 in the NYHA class III group, consistent with prior studies.^[18]^ However, in the age group over 65, while the SF scores remained above 50, they showed a significant decline. The floor effects observed in RP may be explained by the clinical symptoms of SSS, including syncope, fatigue, and palpitations, which can impair work productivity and cognitive function.

The ICC scores calculated for SF-36 showed high test-retest reliability (ICC > 0.70). Internal consistency and split-half reliability are good. The Cronbach alpha coefficients were generally acceptable for group-level comparison in the study. VT and MH, as well as VT and SF, had higher correlations (>0.7) than other scales. Similar findings were also reported by other Asian researchers.^[19]^ High correlations between these subscales may be attributed to cultural differences in which mental health and social connections play a central role in vitality.

CFA results showed that factor 1 covered MH, VT, and SF. This may explain why the mental state, vitality, and social activity are closely associated with the well-being of patients with SSS. Chang et al^[20]^ supported the reorganization of the subscales along the dimensions of well-being and distress, which made the MH and VT items more meaningful. Although cultural differences are likely to influence the understanding of SF-36 questionnaires, the SF-36 indicated good scale validity in the results of the CFA analysis, as well as strong convergent and discriminant validity. Six principal components were identified by the scree plot and were basically consistent with the original 8 dimensions.

Based on available results, Chinese patients with SSS have lower HRQoL than the general population, especially the RP and GH of the SF-36. From Pearson r between the SF-36 subscales and other characteristics, aging and the class of NYHA maintain a linear relationship with most SF-36 subscales. SSS patients have a worsened quality of life with increasing age and a decline in activity. The process of aging is closely linked to the development of SSS and is considered a major risk factor, which is often accompanied by degenerative changes and electrical remodeling dependent on fibrosis. The NYHA symptom class describes the severity of symptoms and exercise tolerance, used to estimate the actual functional ability of a heart failure patient, as well as the measure of risk.^[21]^ In the study, the quality of life for the population tends to decline with age, reflecting SSS deteriorating health with age. Patients with NYHA functional class III have a significantly decreased quality of life. The severity of symptoms and exercise tolerance might contribute to poorer quality of life than aging. Therefore, NYHA could be an important predictor of HRQoL in SSS. These results remind us to focus on the changes in various aspects throughout treatment and follow-up, not only on age.

Although the study has successfully demonstrated that SF-36 had good reliability or validity in assessing the quality of life in patients with SSS, it has certain limitations. First, the questions about personal or household income and the amount and type of activities were not included in the present study, which may be possible to perform a more in-depth analysis. Second, our study did not include pacemaker patients. Future studies might include the pacemaker population for further assessment. Third, participants were recruited at a single hospital, which might bring a potential for selection bias. Fourth, we did not have NYHA functional class I and IV participants in our sample population, possibly due to the small sample size.

## 6. Conclusions

The SF-36 health-related quality of life questionnaire is well applied to assess the quality of life in SSS patients due to its reliability or validity. SSS patients had lower HRQoL scores on the SF-36. HRQoL and well-being can be affected by aging and activity endurance in SSS patients.

**Table 6:** Sensitivity of the Chinese version of SF-36 in age classes: scores [mean (SD)] (N = 199).

SF-36 subscale	Age < 65 N = 100	Age ≥ 65 N = 99	*P* value	ES
PF	48.10 (7.89)	42.43 (9.76)	<.001	0.64
RP	39.26 (21.42)	28.23 (21.53)	<.001	0.52
BP	47.17 (6.96)	45.91 (7.11)	.21	0.18
GH	39.24 (10.48)	34.57 (11.57)	<.001	0.43
VT	53.06 (9.01)	50.42 (9.17)	.04	0.29
SF	55.01 (10.42)	51.84 (10.20)	.03	0.31
RE	48.50 (19.76)	38.70 (24.21)	<.001	0.45
MH	50.80 (9.23)	49.07 (10.57)	.22	0.18
PCS	42.96 (9.62)	37.30 (10.38)	<.001	0.57
MCS	53.46 (12.30)	50.24 (14.49)	.09	0.24

BP = bodily pain, ES = effect size, GH = general health, MH = mental health, MCS = mental component summary, PF = physical function, PCS = physical component summary, RP = physical role, RE = role emotional, SF = social function, SF-36 = Short Form 36, VT = vitality.

**Table 7:** Sensitivity of the Chinese version of SF-36 in NYHA classes: scores [mean (SD)] (N = 199).

SF-36 subscale	NYHA II N = 181	NYHA III N = 18	*P* value	ES
PF	46.01 (8.50)	37.90 (13.29)	<.001	0.9
RP	35.01 (21.95)	21.35 (20.45)	.01	0.63
BP	46.73 (7.13)	44.61 (6.00)	.22	0.3
GH	37.44 (11.27)	31.72 (9.89)	.04	0.51
VT	52.70 (8.31)	42.15 (11.85)	<.001	1.22
SF	54.45 (9.52)	43.23 (13.43)	<.001	1.14
RE	44.35 (22.06)	36.32 (26.84)	.15	0.36
MH	50.83 (9.09)	40.99 (13.44)	<.001	1.04
PCS	40.77 (10.10)	33.83 (11.28)	.01	0.68
MCS	52.67 (12.79)	43.71 (17.68)	.01	0.68

BP = bodily pain, GH = general health, MH = mental health, MCS = mental component summary, NYHA = New York Heart Association, PF = physical function, PCS = physical component summary, RE = role emotional, RP = physical role, SF-36 = Short Form 36, SF = social function, VT = vitality.

## Acknowledgments

We acknowledge the help of the Cardiovascular Department of Guang’anmen Hospital, China Academy of Chinese Medical Sciences.

## Author contributions

**Data curation:** Yao Chen, YuTong Zhou, Xin'ai Zhang.

**Formal analysis:** Yu Huang.

**Writing – original draft:** Qiaomin Wu.

**Writing – review & editing:** Ruxiu Liu.
